# Looking into the IL-1 of the storm: are inflammasomes the link between immunothrombosis and hyperinflammation in cytokine storm syndromes?

**DOI:** 10.1093/discim/kyac005

**Published:** 2022-09-14

**Authors:** Tara A Gleeson, Erik Nordling, Christina Kaiser, Catherine B Lawrence, David Brough, Jack P Green, Stuart M Allan

**Affiliations:** Division of Neuroscience and Experimental Psychology, School of Biological Sciences, Faculty of Biology, Medicine and Health, University of Manchester, Manchester, UK; Geoffrey Jefferson Brain Research Centre, The Manchester Academic Health Science Centre, Northern Care Alliance NHS Group, University of Manchester, Manchester, UK; Lydia Becker Institute of Immunology and Inflammation, University of Manchester, Manchester, UK; Swedish Orphan Biovitrum AB, Stockholm 112 76, Sweden; Swedish Orphan Biovitrum AB, Stockholm 112 76, Sweden; Division of Neuroscience and Experimental Psychology, School of Biological Sciences, Faculty of Biology, Medicine and Health, University of Manchester, Manchester, UK; Geoffrey Jefferson Brain Research Centre, The Manchester Academic Health Science Centre, Northern Care Alliance NHS Group, University of Manchester, Manchester, UK; Lydia Becker Institute of Immunology and Inflammation, University of Manchester, Manchester, UK; Division of Neuroscience and Experimental Psychology, School of Biological Sciences, Faculty of Biology, Medicine and Health, University of Manchester, Manchester, UK; Geoffrey Jefferson Brain Research Centre, The Manchester Academic Health Science Centre, Northern Care Alliance NHS Group, University of Manchester, Manchester, UK; Lydia Becker Institute of Immunology and Inflammation, University of Manchester, Manchester, UK; Division of Neuroscience and Experimental Psychology, School of Biological Sciences, Faculty of Biology, Medicine and Health, University of Manchester, Manchester, UK; Geoffrey Jefferson Brain Research Centre, The Manchester Academic Health Science Centre, Northern Care Alliance NHS Group, University of Manchester, Manchester, UK; Lydia Becker Institute of Immunology and Inflammation, University of Manchester, Manchester, UK; Division of Neuroscience and Experimental Psychology, School of Biological Sciences, Faculty of Biology, Medicine and Health, University of Manchester, Manchester, UK; Geoffrey Jefferson Brain Research Centre, The Manchester Academic Health Science Centre, Northern Care Alliance NHS Group, University of Manchester, Manchester, UK; Lydia Becker Institute of Immunology and Inflammation, University of Manchester, Manchester, UK

**Keywords:** inflammasome, macrophage activation syndrome, hyperinflammation, immunothrombosis, cytokine storm, interleukin (IL)-1

## Abstract

Inflammasomes and the interleukin (IL)-1 family of cytokines are key mediators of both inflammation and immunothrombosis. Inflammasomes are responsible for the release of the pro-inflammatory cytokines IL-1β and IL-18, as well as releasing tissue factor (TF), a pivotal initiator of the extrinsic coagulation cascade. Uncontrolled production of inflammatory cytokines results in what is known as a “cytokine storm” leading to hyperinflammatory disease. Cytokine storms can complicate a variety of diseases and results in hypercytokinemia, coagulopathies, tissue damage, multiorgan failure, and death. Patients presenting with cytokine storm syndromes have a high mortality rate, driven in part by disseminated intravascular coagulation (DIC). While our knowledge on the factors propagating cytokine storms is increasing, how cytokine storm influences DIC remains unknown, and therefore treatments for diseases, where these aspects are a key feature are limited, with most targeting specific cytokines. Currently, no therapies target the immunothrombosis aspect of hyperinflammatory syndromes. Here we discuss how targeting the inflammasome and pyroptosis may be a novel therapeutic strategy for the treatment of hyperinflammation and its associated pathologies.

## Graphical Abstract

**Graphical Abstract:**
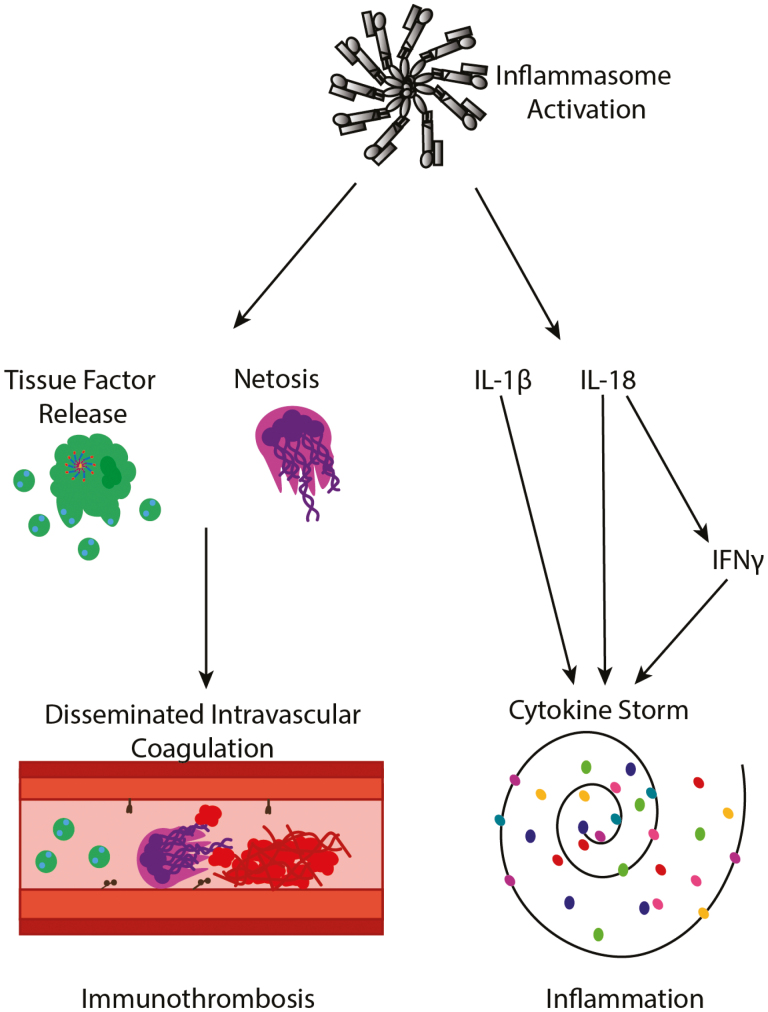

## Introduction

Inflammation is part of an immune response that aims to protect the body from endogenous and exogenous dangers. Inflammation is a direct response to recognition of pathogen-associated molecular patterns (PAMPs) or damage-associated molecular patterns (DAMPs) by their cognate receptors [1, 2]. The ligation of these pattern recognition receptors (PRRs) induces a cascade of cellular events that leads to the production of a variety of cytokines and chemokines, resulting in the activation of immune cells [3]. Depending on the PRR activated, different immune responses can be elicited to contain and eliminate the foreign pathogen, but after clearance of the danger, homeostasis must be restored [4]. However, uncontrolled, or unresolved inflammation can result in a state of hyperinflammation, which leads to tissue damage, organ dysfunction, and dysregulated tissue repair [5]. Hyperinflammation is characterized by the occurrence of a cytokine storm, whereby numerous innate immune cells including macrophages, dendritic cells, and neutrophils act in concert to produce a milieu of inflammatory cytokines which results in tissue damage, creating a positive feedback loop that amplifies inflammation [6].

The term cytokine storm (or hypercytokinemia) encompasses a variety of disorders that involve hyperinflammation and multiorgan dysfunction characterized by excessive release of cytokines [7]. Cytokine storms occur in a variety of different settings including as an aberrant response to infection, immunotherapy, cancers, and autoimmune disorders [7]. Although hyperinflammatory syndromes have been well recognized for many years interest has never been as high as in the wake of the COVID-19 pandemic where induction of a cytokine storm is associated with poor outcomes following SARS-CoV-2 infection [8]. Cytokine storms are associated with rheumatic diseases such as systemic juvenile idiopathic arthritis (SJIA) and the adult form of the disease, adult onset Still’s disease (AOSD), where periods of overwhelming inflammation are often described as macrophage activation syndrome (MAS), which is characterized by excessive production of systemic cytokines accompanied by the expansion and activation of T cells and by hemophagocytic macrophages [6, 9]. Another disease classified as a cytokine storm syndrome is hemophagocytic lymphohistiocytosis (HLH). There are variations of HLH, with primary HLH (pHLH) mediated by genetic defects and secondary HLH (sHLH) driven by environmental factors [10], with both exhibiting similar clinical features to MAS. Cytokine storms are also present in cytokine release syndrome, which occurs in response to severe bacterial infection and sepsis [11], or from drugs, in particular in response to antibody-based therapies [12]. The role of cytokine storms in severe bacterial infection and sepsis has previously been extensively reviewed [11]. Despite the diversity of signals that induce cytokine storms, hyperinflammatory diseases often share clinical symptoms including overwhelming systemic inflammation, multiorgan system disease, hemophagocytic macrophages, coagulopathies, immunothrombosis, and often result in death [13]. At present, the criteria for diagnosis of cytokine storm syndromes/hyperinflammation are not consistent given the heterogeneity of disease. Though the diagnosis of cytokine storm is difficult, a number of clinical parameters are documented to change in patients with MAS including, decrease in platelet count, elevated serum levels of ferritin, lactate dehydrogenase, and aspartate aminotransferase [14]. Since cytokine storm is not a diagnosis of exclusion, it is difficult to differentiate between sepsis, disease flare-ups or MAS, delaying treatment and ultimately contributing to the poor outcomes associated with cytokine storm [14]. As a result, a unifying definition for cytokine storm syndromes was recently proposed to assist with diagnosis, classifying cytokine storm in patients with elevated circulating cytokine levels, acute systemic inflammation, and secondary organ dysfunction beyond a normal response to a pathogen (in circumstances of pathogen mediated hyperinflammation) [7].

Coagulopathies and immunothrombosis are implicated in the pathogenesis of cytokine storm syndromes and contribute to lethality, in particular these phenomena have been noted in patients with SARS-CoV-2 infections [15]. Immunothrombosis describes the interplay between the innate immune system and coagulation cascade that leads to thrombus formation [16, 17]. The extensive cross-talk between the two systems ultimately aims to provide host defence; however, dysregulation of the immune system can result in hyperactivation of the coagulation cascade, and vice versa [18]. Dysregulation of the coagulation cascade is observed in hyperinflammatory syndromes, and thrombosis-associated disseminated intravascular coagulation (DIC) carries a high burden of mortality, thus highlighting the need for novel therapeutic targets.

In this review, we address the relationship between cytokine storm and thrombosis in hyperinflammatory disease and discuss the potential role of inflammasomes and interleukin (IL)-1 cytokines as critical mediators and therapeutic targets.

## Cytokine storm and hyperinflammatory disease

Initiation and perpetuation of cytokine storms is primarily mediated by innate immune cells such as macrophages, neutrophils, and natural killer (NK) cells [6]. Macrophages exposed to a cytokine storm adopt an extremely inflammatory phenotype and drive an uncontrolled inflammatory response through the further secretion of cytokines resulting in the development of hyperinflammatory disease [19]. At present, the knowledge of key cytokines involved in the storm is largely derived from current models of HLH and MAS. While multiple proinflammatory cytokines are upregulated in these disorders, the dominant cytokine driving disease observed in both animal models of MAS/HLH and patients with HLH is interferon-γ (IFNγ) [20–23]. Administration of IFNγ monoclonal antibodies to the mouse models of pHLH [21] and MAS [21, 23] reduces disease severity. In pHLH genetic defects in CD8^+^ T cells and NK cells impair cytotoxic function, and inability to clear pathogens is thought to induce hyper-production of IFNγ in this disease [24, 25]. However, use of Ruxolitinib, a Janus Kinase (JAK) inhibitor, significantly reduced hypercytokinemia, splenomegaly, and death in an animal model of sHLH to a greater extent compared with administration of IFNγ monoclonal antibodies [26]. It is hypothesized that ruxolitinib is capable of ameliorating disease since a variety of the cytokines implicated in cytokine storm signal through JAK1/2 or are induced by cytokines that signal via the JAK-STAT pathway, suggesting targeting IFNγ alone may be insufficient to fully control cytokine storms [27].

While IFNγ signalling is crucial for development of hyperinflammatory disease, there is increasing evidence additional cytokines are important in cytokine storm development. Stimulation of bone marrow-derived macrophages (BMDMs) with a cocktail of cytokines indicates that IFNγ and TNF play fundamental roles in the induction of hyperinflammation *in vitro* [28]. Administration of recombinant cytokines including IL-1, IL-6, IL-12, IL-18, and TNF can induce lethal hyperinflammation similar to that witnessed in cytokine storms [7], highlighting the complexity of the factors involved in cytokine storms. Given that hyperinflammation is induced in both animals and humans in response to recombinant cytokine therapy, it is no surprise that monoclonal antibody therapy targeting the pro-inflammatory cytokines IL-1β, IL-6 and TNF all reduce symptoms and storm severity [29, 30], highlighting the potential benefits of various forms of anti-cytokine therapy in treating hyperinflammatory disease. The IL-1 cytokine family present a particularly intriguing target for treating hyperinflammatory diseases given their role as early cytokines, in that they drive the transcription of numerous downstream inflammatory processes, including the induction of IFNγ [31]. The IL-1 superfamily is composed of eleven cytokines; IL-1α, IL-1β, IL-1 receptor agonist (IL-1Ra), IL-18, IL-33, IL-36α, IL-36β, IL-36γ, IL-36Ra, IL-37, and IL-38 [31] that have different functions (as reviewed [32]). Though there are numerous proinflammatory IL-1 family cytokines, the focus in this review is the inflammasome and inflammasome-dependent cytokines, and their roles in bridging the gap between cytokine storm and immunothrombosis.

**Figure 1:**
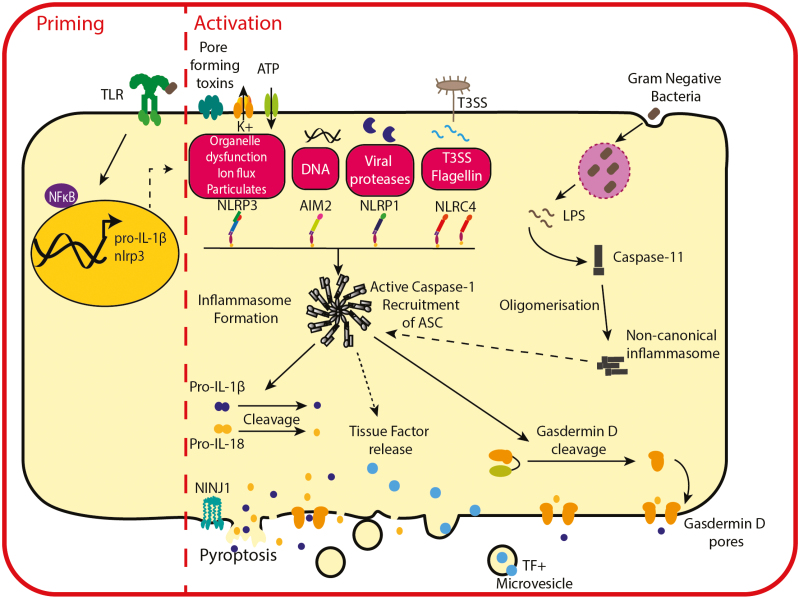
The inflammasomes. Prior to inflammasome activation, priming stimuli, such as TLR ligands, drive the NF-κB-dependent expression of pro-IL-1β. Inflammasomes are formed in response to a wide variety of PAMPs/DAMPs activating distinct inflammasome PRRs, such as NLRP3, AIM2, NLRP1, and NLRC4. Despite containing different PRRs, formation of the inflammasome results in the same downstream caspase-1-dependent cleavage of pro-IL-1β, pro-IL-18, and gasdermin D (GSDMD). GSDMD pores are inserted into the plasma membrane, facilitating release of IL-1β and IL-18, and NINJ1 oligomerisation occurs resulting in pyroptosis. The non-canonical inflammasome targets caspase-4/5 in humans (caspase-11 in mice), which can directly recognize microbial products such as LPS, triggering pyroptosis through the cleavage of GSDMD. Potassium (K+) efflux through GSDMD pores can lead to NLRP3 activation and subsequent IL-1β and IL-18 processing. Activation of inflammasomes also results in TF release and activation of coagulation system. TLR, Toll-like receptor; PAMP, pathogen-associated molecular pattern; DAMP, damage associated molecular pattern; PRR, pattern recognition receptor; TF, tissue factor.

The most relevant IL-1 family members in propagating cytokine storm syndromes are the potent upstream IL-1 cytokines (IL-1α and IL-1β) and IL-18, release of IL-1β and IL-18 being dependent on activation of inflammasomes (Fig. 1). Inflammasomes are multimolecular protein complexes containing a sensor PRR, the adaptor protein ASC (apoptosis-associated speck-like protein containing a CARD), and the protease caspase-1 [33]. Upon formation of the inflammasome, caspase-1 recruitment leads to its auto-proteolytic activation resulting in two distinct events: (i) the cleavage of IL-1β and IL-18 into their active forms and (ii) the cleavage of the pore forming protein gasdermin-D (GSDMD) which allows for release of mature IL-1β and IL-18 [33]. Plasma membrane rupture is mediated by NINJ1 protein clustering which induces a highly inflammatory form of cell death known as pyroptosis and release of cytosolic contents and DAMPs [34]. Numerous inflammasome complexes have been defined, which vary by their scaffolding PRR, including NLR (Nod-like receptor) family pyrin domain containing (NLRP)1, NLRP3, NLR family CARD domain-containing protein 4 (NLRC4), absent in melanoma 2 (AIM2), and the non-canonical caspase-4/5 (caspase-11 in mice) inflammasome [35]. Each inflammasome PRR is activated by different ligands or cellular stresses leading to inflammasome activation in response to a range of diverse threats [35] (Fig. 1). IL-1α is released in response to rapid calcium influx following necrotic cell death, including following inflammasome activation [36]. Like IL-1β and IL-18, IL-1α is synthesized in a precursor form but is cleaved to mature IL-1α by calcium activated proteases, such as calpains rather than caspases [36].

### IL-1 and IL-18 in hyperinflammation

**Figure 2:**
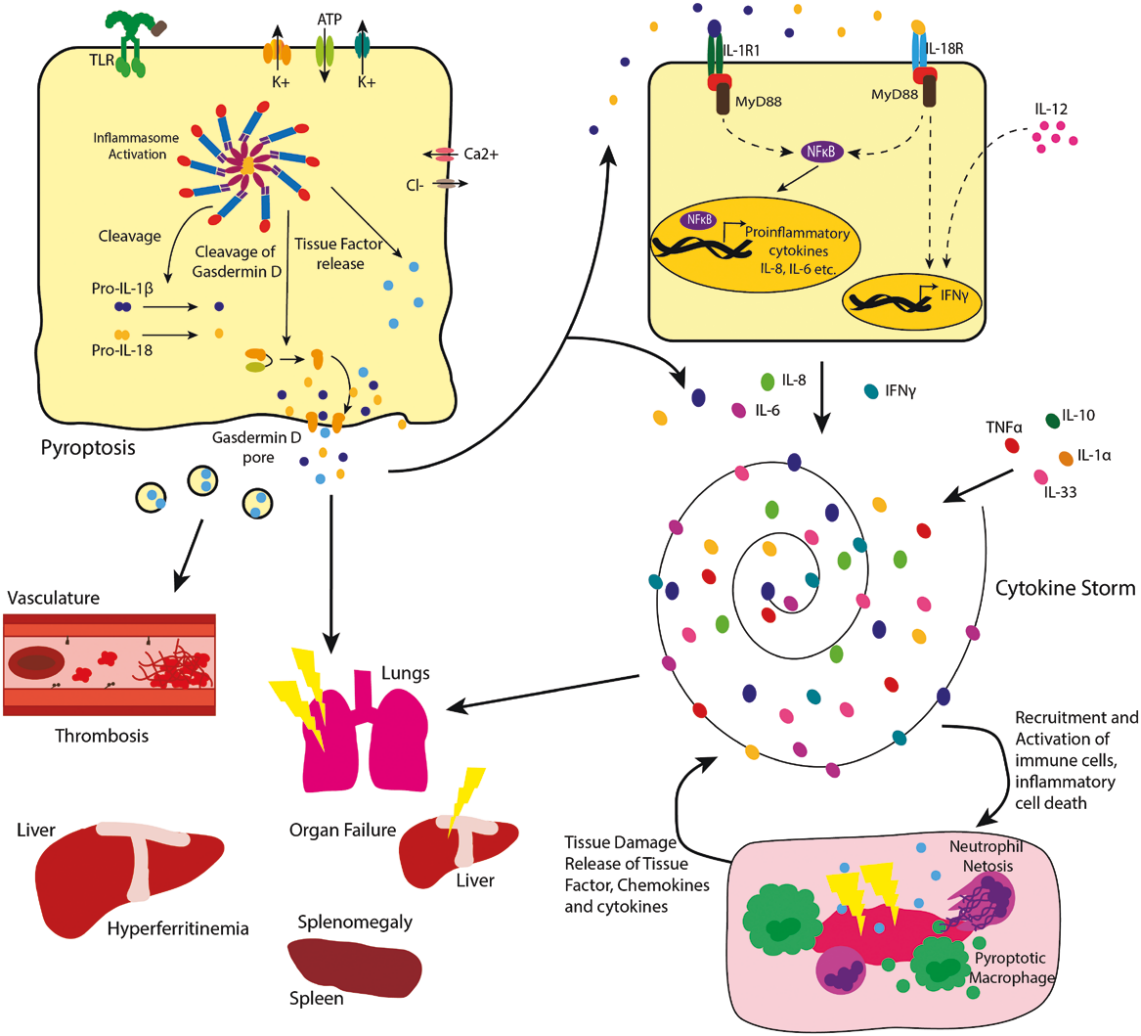
Inflammasome activation contribute to cytokine storm development and hyperinflammation. Activation of the inflammasome results in release of mature IL-1β and IL-18, which signals via the IL-1 receptor (IL-1R1) and IL-18 receptor (IL-18R) on target cells. IL-1R1 and IL-18R signalling drive NF-κB signalling leading to the expression of pro-inflammatory genes and cytokines such as TNF, IL-6 and IL-8. IL-18, along with IL-12 have a key role in driving an interferon-γ response, a cytokine that is crucial for hyperinflammatory disease such as MAS. This highly inflammatory environment mediated by uncontrolled cytokine release is known as a cytokine storm. Uncontrolled activation of the immune system results in augmented recruitment of immune cells to the site of inflammation resulting in tissue damage and further release of DAMPs and tissue factor therefore amplifying cytokine storms, leading to characteristic pathology such as hyperferritinaemia, splenomegaly and microthrombi.

IL-1 and IL-18 are key contributors to the induction of hyperinflammation (Fig. 2). The importance of IL-1 in driving cytokine storm syndromes is seen through the beneficial effects of recombinant IL-1Ra (anakinra) in ameliorating hypercytokinemia in COVID-19 and SJIA complicated with MAS [37, 38]. Anakinra is licensed by the European Commission (EC) for the treatment of several hyperinflammatory diseases, such as SJIA and is recommended for use off-label in the “2021 American College of Rheumatology Guideline for the Treatment of Juvenile Idiopathic Arthritis” [38]. Children with SJIA complicated by MAS respond better to anakinra treatment rather than anti-TNF therapy indicating the critical importance of IL-1 family cytokines in driving MAS [39]. Anakinra treatment also reduced symptoms in patients with MAS who were previously unresponsive to corticosteroid treatment, however the approved dose of anakinra is often insufficient to fully resolve the hyperinflammatory state [40, 41]. Furthermore, as a potential therapy for MAS, anakinra has a favourable non-myelosuppressive safety profile, compared with other treatments trialled for use in cytokine storm syndromes such as etoposide, tocilizumab and ruxolitinib [37]. However, anakinra does not seem to fully protect all individuals from MAS development, as in rare circumstances, patients receiving anakinra treatment for SJIA/AOSD can still progress to MAS [41].

Not only is anakinra treatment beneficial in cytokine storm syndromes, but clinical data also further support IL-1 driving disease phenotype. Peripheral blood mononuclear cells (PBMCs) from patients with SJIA are highly inflammatory and release considerable quantities of IL-1β when activated with PMA-ionomycin [42]. Pathway analysis corroborates IL-1β as a key mediator of SJIA flares, with IL-1β identified as the central molecular driver altering protein expression and propelling a hyperinflammatory phenotype [43]. In murine models of MAS, plasma IL-1β levels are elevated along with a variety of cytokines such as IL-6, IL-10, and IL-18 indicating cytokine storm development [44]. Further recent evidence of the role of inflammasomes in driving MAS and hypercytokinemia comes from studies in COVID-19 (see review [45]). Characterization of serum cytokines present in patients with COVID-19 demonstrates elevated levels of TNF and IL-6 [46], and IL-1Ra completely abrogates IL-6 and TNF release from SARS-CoV-2-infected primary human monocytes [47]. Recent studies demonstrate activation of the NLRP3 and AIM2 inflammasome in monocytes and macrophages in SARS-CoV-2-infected patients [48, 49]. Further in rare circumstances, COVID-19 mRNA vaccination induces multisystem inflammatory syndrome in children [50], which is dependent on inflammasomes and IL-1 cytokines, and is prevented by IL-1 blockade [51]. However, clinical data on blockade of IL-1 signalling in COVID-19 is inconclusive with limited effects of treatment on mortality [52–54].

In comparison to Anakinra, which blocks both IL-1α and IL-1β signalling, less research has been gathered on the use of anti-IL-1β monoclonal antibodies (canakinumab) and IL-1β receptor decoy antibodies (rilonacept) in cytokine storm syndromes. Some data suggest that canakinumab may be efficacious in treatment of hyperferritinemic syndromes [55]; however, this is contradicted with instances of SJIA patients developing MAS while on canakinumab therapy [30, 56, 57]. Clinical trials examining the efficacy of canakinumab treatment in severe COVID-19 revealed limited increase in survival of patients compared with placebo [53] (Clinicaltrials.gov identifier NCT04362813). Similarly, use of rilonacept, an IL-1β receptor decoy, appears efficacious in managing active SJIA (without MAS); however, its ability to modulate hyperinflammatory syndromes has not been examined [58].

Interestingly, despite the efficacy of anti-IL-1Ra therapies in treatment of hyperinflammatory diseases, excessive IL-1 signalling alone appears to be insufficient to cause development of cytokine storms. This is best demonstrated in conditions where genetic mutation in inflammasome components lead to aberrant IL-1 and IL-18 signalling, including familial mediterranean fever, cryopyrin-associated periodic syndrome, and neonatal-onset multisystem inflammatory disease (NOMID) [59]. While some symptoms are similar between MAS and these inflammasomopathies, such as fever [14, 60], other symptoms are distinct. For example, MAS commonly presents with reduced platelet and leukocyte counts, hyperferritinaemia, splenomegaly, and microthombosis that are absent in the majority of reported genetic inflammasomopathies [61, 62]. Despite such differences in symptoms there is still good evidence supporting a key role of IL-1 family cytokines in the development of cytokine storms.

IL-18 is emerging as a critical cytokine in hyperinflammatory disease with high levels seen in both human and murine manifestations of MAS [62, 63]. Circulating blood levels of IL-18 are consistently higher in patients with AOSD or SJIA with active MAS than those without MAS or in other inflammatory rheumatic diseases without cytokine storm, such as rheumatoid arthritis and systemic lupus erythematous [63–65], suggesting that IL-18 may drive a cytokine storm rather than just rheumatic inflammation. IL-18 levels correlate with cytokine storm markers such as C-reactive protein, ferritin, neopterin, IL-6, and soluble TNFR1 levels [64]. High levels of IL-18 in the serum of patients with hyperinflammation correlate with both Th1 immune cell markers and macrophage activation [66]. There also appears to be a significant negative correlation between the level of free IL-18 and the NK cell count, indicating a possible mechanism through which IL-18 may be modulating the activation of immune cells in the context of HLH/MAS [66]. Other markers associated with disease also correlated with IL-18 concentrations including anaemia and ferritineamia [62].

As mentioned earlier, MAS is rarely observed in inflammasomopathies, but notably a missense mutation in the NLRC4 inflammasome (Thr337Ser) results in enhanced NLRC4 activity and autoinflammation leading to recurrent MAS [67]. Patients with NLRC4 Thr337Ser mutations exhibit a substantial increase in plasma IL-18 to levels comparable to other instances of MAS flares in SJIA, AOSD, and infection, which is not present in non-MAS inflammasomopathies such as NOMID [67, 68]. Furthermore, macrophages isolated from patients with NLRC4 mutations (Thr337Ser) release more IL-18 upon activation than macrophages from patients with NOMID, suggesting that the enhanced IL-18 signalling is contributing to cytokine storms and the development of MAS [67].

The IL-18 receptor is most frequently expressed on CD8^+^ T cells, playing a crucial role in the production of IFNγ [69, 70] which is strongly implicated in pHLH. In murine models of MAS, the reciprocal relationship between IL-18 and IFNγ is evident, with both cytokines required for the development of a cytokine storm and the removal of one impacts the perpetuation of the other leading to recovery of hyperinflammation [44]. Incubation of a macrophage cell line with serum samples from patients with active AOSD results in the induction of IFNγ production, indicating the levels of IL-18 in the serum are sufficient to induce IFNγ production and can be reversed through administration of IL-18 binding protein (BP), an endogenous regulator of IL-18 [71, 72]. Uncontrolled IL-18 signalling in IL-18BP knockout (IL-18BP^-/-^) mice results in exacerbated manifestations of MAS [73]. In addition to more severe weight loss, splenomegaly, thrombocytopenia, hyperferritinaemia, and bone marrow hemophagocytosis, IL-18BP^−/−^ mice also have significantly increased circulating IFNγ levels, further supporting a role for IL-18 in driving IFNγ release and ultimately MAS [73]. Importantly, blockade of either IL-18 receptor or IFNγ signalling alleviates MAS severity compounding the role of reciprocal regulation of IL-18 and IFNγ in MAS [73].

Since IL-18 has been implicated in MAS development, strategies to limit IL-18 signalling in hyperinflammatory diseases have been tested as potential therapeutics. IL-18BP is a potent inhibitor of IL-18, by sequestering released IL-18 and preventing signalling at the IL-18 receptor, and can be a useful therapeutic to limit IL-18 responses in hyperinflammatory disease [68, 74, 75]. Patients with mutations in the NLRC4 inflammasome leading to MAS treated with recombinant IL-18BP (Tadekinig alfa) display an alleviation in MAS symptoms and a drastic reduction in circulating cytokines and ferritin [68] with further phase III clinical trials underway (Clinicaltrials.gov Identifier: NCT03113760).

Despite the evidence that IL-1β and IL-18 are key contributors to the development of cytokine storm syndromes, there has been little research on which inflammasomes are activated in the majority of hyperinflammatory diseases, though recent data has been obtained in severe COVID-19 [45], with studies demonstrating activation of the NLRP3 inflammasome in monocytes and macrophages in SARS-CoV-2-infected patients [48, 49]. It is hypothesized that inflammasomes are crucial in driving the pathology associated with severe COVID-19, since inflammasome activation leads to maturation of IL-1β and IL-18, along with the formation of pores in the cell membrane leading to the release of tissue factor (TF) positive microvesicles and netosis in neutrophils, both of which contribute to coagulation in the vasculature (as reviewed [45]).

## Immunothrombosis

Coagulation is the process through which a blood clot is formed and plays an important role in the prevention of excessive bleeding in the case of a wound, but also serves as an evolutionarily conserved mechanism of containing foreign pathogens [76]. DIC, the excessive formation of blood clots, is a consequence of systemic activation of the coagulation cascade [77]. Ultimately, the unbalanced communication between the immune system and the coagulation cascade in hyperinflammatory diseases with cytokine storm often results in the development of DIC and multiorgan failure (as reviewed [78]). Inflammasomes play a crucial role in inflammation and contribute to immunothrombosis through a variety of mechanisms. Upon formation of the inflammasome, caspase-1 processes pro-IL-1β and pro-IL-18 to their mature, functional forms while also cleaving GSDMD resulting in oligomerization and pore formation contributing to pyroptosis [79]. These processes of cytokine release, pore formation, and inflammatory cell death can contribute to clot formation, fuelling speculation that inflammasomes promote coagulopathies in cytokine storm syndromes.

**Figure 3:**
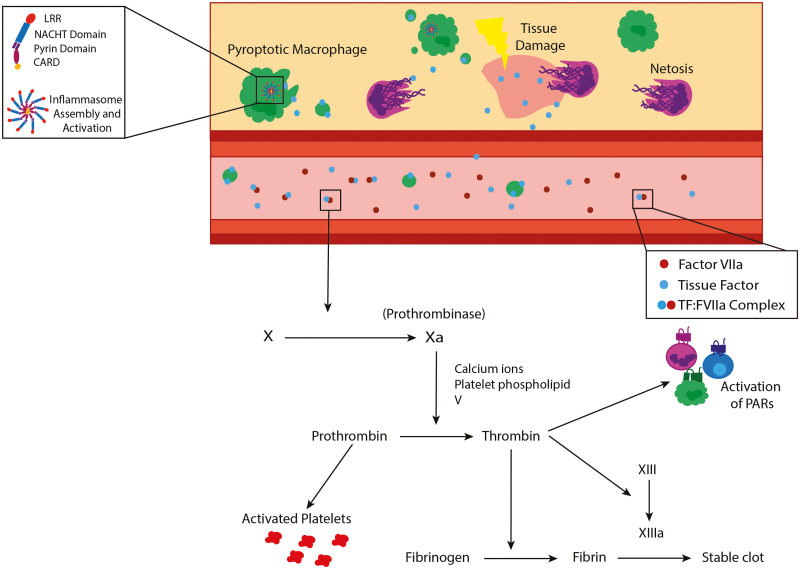
The extrinsic coagulation cascade promotes clotting to tissue damage. Damage to blood vessels or exposure of collagen drives activation of the intrinsic coagulation cascade. Tissue damage caused by inflammation, injury, or infection drives the release of tissue factor driving initiation of the extrinsic coagulation cascade. TF and factor VIIa colocalize in the bloodstream to form the TF-factor VIIa complex that can drive activation of PARs on the surface of immune cells. The TF-factor VIIa complex also drives activation of factor X, similarly in the intrinsic pathway coagulation factors converge on the activation of factor X. Ultimately, all coagulation pathways converge on the conversion of factor X to its active form which in combination with calcium ions, platelet membrane phospholipid, and factor V lead to the cleavage of pro-thrombin to thrombin. Thrombin carries out a variety of functions including activation of immune cells via PARs, cleavage of fibrinogen to fibrin, and activation of factor XIII. Active factor XIII and fibrin are needed to form a stable fibrin clot. TF. tissue factor, PAR, protease activating receptor.

TF (also known as factor III) is perhaps the most important of the glycoproteins in the coagulation cascade in response to injury and infection. TF is the primary initiator of the extrinsic coagulation cascade (Fig. 3 and summarized [80]) and is released from damaged tissue, or can be upregulated by inflammation, sepsis, and hypoxia [81]. Ultimately, TF release leads to processing of pro-thrombin to active thrombin, a serine protease, which then catalyses the conversion of fibrinogen to fibrin, leading to the formation of the crosslinked fibrin, providing a scaffold for the formation of clots (Fig. 3) [18]. Inflammasome assembly and activation are known to play a crucial role in sepsis associated thrombus formation. Mouse models of septicaemia exhibit inflammasome activation and pyroptosis leading to the release of TF-rich extracellular vesicles resulting in DIC and lethality [82]. LPS-induced coagulation and lethality is reduced by pre-depletion of monocytes and macrophages, indicating that these cells are crucial sources of TF, and by genetic deletion of caspase-1/11 or GSDMD [82], highlighting an essential role of inflammasomes in TF release. The incidence of venous thrombus in response to inferior vena cava stenosis is also significantly reduced in mice deficient in either GSDMD or caspase-1 [83] and hypoxia-induced thrombosis is reduced by treatment with NLRP3 siRNA or caspase-1 inhibition [84]. Furthermore, activation of the NLRP3 inflammasome induces release of TF in phosphatidylserine-positive microparticles in cultured macrophages [85]. Murine models of sepsis also demonstrate roles for TLR4 and the non-canonical inflammasome (mouse caspase-11, human caspase-4/5) in flipping phosphatidylserine (PS) to the outer cell membrane contributing to the coagulation cascade. Under homeostatic conditions, PS is localized to the inner leaflet of the plasma membrane as well as in endocytic vesicles; however, during sepsis, PS becomes expressed on the cell surface of circulating leukocytes, contributing to the formation of clots [86, 87]. The expression of PS on the cell surface facilitates TF decryption, whereby TF becomes active and can partake in the coagulation cascade [86]. In essence, the activation of multiple inflammasomes have been linked to TF release, through GSDMD pores or pyroptosis, resulting in DIC. Targeting the inflammasome reduces the incidence of DIC in animal models indicating that inhibition of the inflammasome or pyroptosis may be a viable target in treating immunothrombosis.

Not only are inflammasomes themselves responsible for altering release of TF by GSDMD-mediated pyroptosis, the release of IL-1β, IL-18, and downstream inflammatory cytokines is also capable of influencing TF expression. There are several cytokines capable of altering TF expression such as TNF, IL-1α, IL-1β, IL-6, IL-8, and IFNγ [78]. Upregulation of TF on the surface of cells by inflammatory cytokines increases the likelihood of TF activation and initiation of the coagulation cascade therefore creating an inflammatory feedback loop like what is expected to occur in cytokine storm syndromes.

### Inflammasomes and netosis

Neutrophils play a central role in promoting the development of cytokine storms and immunothrombosis. Platelets and neutrophils are quickly recruited to the sites of inflammation or endothelial vessel damage [88] to prevent uncontrolled bleeding and invasion of pathogens. However, like inflammation, uncontrolled activation and recruitment of platelets and neutrophils can result in the formation of a pathologic thrombus. Netosis is a form of inflammatory cell death in neutrophils, which results in the release of a variety inflammatory stimuli, including neutrophil extracellular traps (NETs). The release of NETs from neutrophils provides a scaffold for the recruitment and activation of platelets and plasma proteins such as von Willebrand factor, fibronectin, and fibrinogen [89]. In addition to providing a scaffold for procoagulants, neutrophils themselves can be a source of factor XII, a key component of the intrinsic coagulation cascade [90]. Neutrophil-derived factor XII also promotes netosis, thus creating an inflammatory feedback loop, whereby the release of NETs results in tissue damage and further release of extrinsic coagulation factors such as TF [91]. The ability of fibrinogen to directly bind to NETs indicates the importance of NETs in driving clot formation, as well as the neutrophil’s ability to produce prothrombotic factors implies the key function of neutrophils in linking the immune and coagulation systems.

Inflammasomes may also contribute to immunothrombosis in hyperinflammatory syndromes by promoting the release of NETs [92]. As well as facilitating pyroptosis in monocytes and macrophages, GSDMD has an essential function in co-ordinating netosis of neutrophils [93, 94]. Neutrophils have functional inflammasomes, such as NLRP3, NLRC4, and AIM2, but can resist pyroptosis following inflammasome activation through reduced cleavage of GSDMD, leading to sub-lytic GSDMD pore formation [95]. However, activation of the non-canonical inflammasome via caspase-11 results in robust GSDMD cleavage and results in netosis, rather than pyroptosis [96]. GSDMD is also cleaved by serine proteases other than caspase-11 to trigger netosis, such as neutrophil elastase [93, 97]. Further investigation is needed to identify whether inflammasomes in neutrophils contribute to netosis in hyperinflammation, since targeting GSDMD could be an effective strategy to limit netosis and DIC.

### Inflammasome activation in platelets

In addition to TF release, inflammasomes may also play a critical role in the activation of platelets leading to thrombus formation. Although the mechanisms behind NLRP3 inflammasome activation in platelets is yet to be fully characterized, platelets treated with NLRP3 inhibitors or from NLRP3-deficient mice show significantly reduced platelet activation in response to collagen [98]. Further to platelet activation, NLRP3-deficient platelets have significantly less thrombus area than their WT counterparts [98]. *In vivo* NLRP3-deficient mice appear to have impaired haemostasis and arterial thrombosis, indicating a crucial role for NLRP3 in mediating the link between immunity and thrombosis [99]. *In vitro* use of the NLRP3 inhibitor CY-09 leads to a significant reduction in aggregation of human platelets. Intriguingly, the addition of recombinant IL-1β to NLRP3-deficient platelets rescued the defect in clot formation and restored clot retraction, suggesting that platelet-derived IL-1β is a key driver in blood clotting [99]. In this way, the NLRP3 inflammasome plays a crucial role in mediating haemostasis and clot formation and perhaps activation of the NLRP3 inflammasome in platelets in hyperinflammation results in the associated immunothrombosis. In addition, the presence of platelets enhances inflammasome responses in human macrophages, monocytes, and neutrophils [100], indicating that interaction between blood and elements of clots modifies the innate immune response, further highlighting the cross-talk that exists between the immune and coagulation systems.

### Inflammation drives immunothrombosis and vice versa

Not only does inflammation drive thrombosis, but thrombosis itself can cause inflammation therefore creating a feedback loop that contributes to the manifestation of hyperinflammatory syndromes complicated by thrombosis. Cleavage of fibrinogen by thrombin during the coagulation cascade results in activation of IL-1α and IL-1 signalling [101]. Macrophages, keratinocytes, and platelets express pro-IL-1α (p33) on their surface [101]. Pro-IL-1α can be activated by both calpain and thrombin, with calpain cleaving pro-IL-1α (p33) to a mature IL-1α (p17). However, thrombin differentially cleaves p33 at a highly conserved PRS motif to give an 18 kDa fragment (p18) [101]. Both cleavage sites result in active forms of mature IL-1α. Activation of IL-1α by thrombin leads to rapid thrombopoiesis and wound healing [101]. p18 has also been shown to be generated in humans during sepsis, indicating that IL-1α plays a pivotal role in linking inflammation and coagulation [101].

IL-1α is essential for maintaining haemostasis in response to injury as activation of IL-1α results in rapid thrombopoiesis via rupture of megakaryocytes. Upon exposure to IL-1α megakaryocytes become irregular in shape followed by release of platelet-like particles. These platelet-like particles enter the bone marrow blood vessels and can then travel to sites of damage to prevent bleeding and encourage wound healing [102]. Interestingly, the levels of thrombopoietin, a key hormone produced by the liver and kidneys responsible for platelet production, are not elevated in response to acute platelet loss indicating that IL-1α provides a rapid release of platelets in response to thrombocytopenia [102]. Similarly, administration of an IL-1R antibody reduced platelet production. Therefore, it is possible that high levels of IL-1α in the context of hyperinflammation may be sufficient to drive rupture of megakaryocytes aiding in the formation of blood clots in the vasculature.

## Discussion

**Figure 4:**
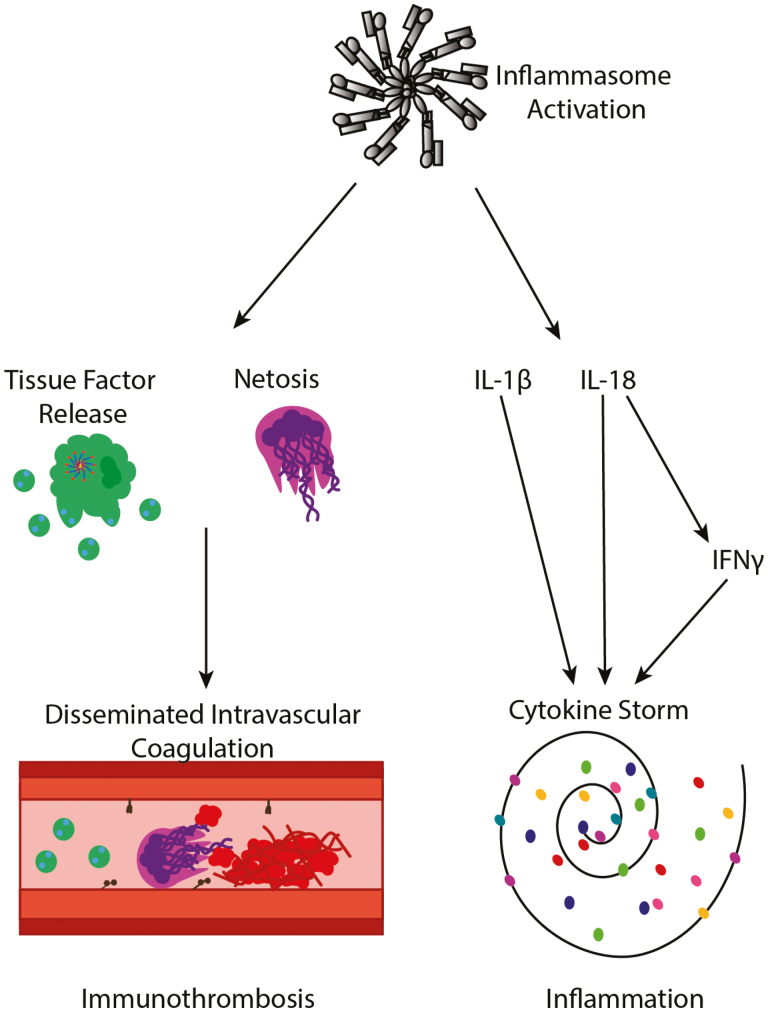
Inflammasomes are poised as key regulators of both immunothrombosis and inflammation. Activation of the inflammasome within monocytes leads to inflammatory cell death and release of TF-positive microvesicles. Activation of the inflammasome in neutrophils induces netosis, an inflammatory form of cell death whereby nuclear contents are thrust out of the cell forming neutrophil extracellular traps (NETs). These inflammasome-dependent cellular death events lead to the formation of thrombi in the vasculature, a phenomenon known as disseminated intravascular coagulation (DIC). Similarly, activation of inflammasomes leads to the release of the pro-inflammatory cytokines IL-1β and IL-18. IL-18 is of particular importance in cytokine storms as it promotes production of IFNγ in T cells, which is a crucial cytokine in MAS/HLH. In addition to enhancing the production of IFNγ, IL-1β and IL-18 act on downstream cytokines leading to the production of a variety of cytokines and contributing to the pathology of cytokine storms.

Hyperinflammation-associated coagulopathy exemplifies the depth of interaction that exists between the immune system and the coagulation cascade. A procoagulant state is formed in response to hyperinflammation through activation of inflammasomes resulting in release of potent proinflammatory cytokines that potentiate cytokine storms and activate a variety of procoagulant responses, including activation of platelets, netosis, and release of TF-positive microvesicles, and the expression of phosphatidyl serine on the external surface of the cell membrane, leading to clot formation (Fig. 4). The ability of inflammasomes to promote immunothrombosis poises them as key regulators of this phenomenon.

Although hyperinflammation is not a new phenomenon, research into the area of MAS has been limited, but slowly we are gaining a better understanding of the factors at play in driving cytokine storms. Inflammasomes appear to be crucial regulators of cytokine storms and associated thrombosis indicating a novel therapeutic target for hyperinflammatory syndromes. Off-label use of anakinra in cytokine storm syndromes, including both COVID and SJIA and the phase III clinical trial for Tadekinig alfa for NLRC4-MAS provides increasing clinical evidence supporting the importance of inflammasome-dependent cytokines in driving hyperinflammatory syndromes. Given the incidence of thrombotic events in hyperinflammatory syndromes and the ability of inflammasome activation to drive a thrombotic response, it appears as though inflammasome activation is the point whereby hyperinflammation can be linked to thrombosis. For this reason, direct inhibition of the inflammasome may be a beneficial treatment option for patients with cytokine storm-induced MAS as it will prevent pyroptosis, therefore preventing the release of DAMPs that propagate cytokine storms, while also preventing maturation of IL-1β and IL-18, which drive an array of downstream cytokine responses as well as having an impact on the formation of blood clots. Presently, there are a variety of NLRP3, caspase-1 and GSDMD inhibitors being investigated for use in inflammatory diseases, with many showing promise in early stage clinical trials (as reviewed extensively in [103]). Therefore, targeting specific inflammatory pathways such as inflammasomes may represent a feasible and effective therapy to prevent serious clotting events in hyperinflammatory diseases by preventing both cytokine storms and immunothrombosis.

## Data Availability

Data sharing not applicable to this article as no datasets were generated or analysed during this paper.
